# Effects of Injury Registry Data on Policy Making, Hospitalizations, and Mortality: Protocol for a Systematic Review and Meta-Analysis

**DOI:** 10.2196/55029

**Published:** 2024-10-30

**Authors:** Ana Cláudia Medeiros-de-Souza, Luana Emanuelly Sinhori Lopes, Bruno Zocca de Oliveira, Edna Terezinha Rother, Lucas Reis Correia

**Affiliations:** 1 Hospital Israelita Albert Einstein Sao Paulo Brazil; 2 Department of Preventive Medicine University of São Paulo São Paulo Brazil

**Keywords:** injury registry, trauma registry, policy making, health policy, wounds and injuries, outcome assessments, health surveillance, hospitalizations, mortality

## Abstract

**Background:**

Initiated in 2021, a Brazilian project aims to establish a national injury registry, compiling comprehensive data on events and individuals across the country, irrespective of injury severity. The registry integrates information from prehospital and hospital care, diverse health systems lacking interoperability, and sectors such as firefighters and the police. Its primary goal is to enhance health surveillance by providing timely, high-quality information, guiding prevention strategies, and informing policy making. The project still aims to reduce long-term morbidity and mortality associated with injuries.

**Objective:**

A knowledge gap remains regarding the effects of injury registries in relation to policies and injury outcomes. This protocol outlines a systematic review and meta-analysis to answer “What is the effect of implementation and use of injury registry data on policy making, hospitalization, and mortality?”

**Methods:**

The systematic review follows PRISMA (Preferred Reporting Items for Systematic Reviews and Meta-Analyses) guidelines, focusing on studies reporting results related to the implementation and use of injury registries, including trauma registries. Outcomes of interest include policy making, hospitalization rates or duration, and mortality. Registries within well-defined administrative boundaries will be included. Data will be collected from PubMed, Embase, Scopus, Web of Science, Lilacs, and references. Records will be independently screened by 2 reviewers, with any disagreements resolved through arbitration by a third reviewer. Homogeneous studies, with 3 or more evaluating the same outcome, may undergo meta-analysis. Subgroup analyses by registry type, injury groups, and other selected variables of interest will be conducted. Sensitivity analysis, risk of bias assessment, publication bias evaluation, and quality appraisal will also be performed.

**Results:**

This systematic review will run from November 2023 to June 2024. No identical review was found. Search strategies were finalized, the bibliographic search started, duplicates were eliminated, and title and abstract screening began. Of 35 studies retrieved, 85 were excluded due to duplication, leaving 50 for selection.

**Conclusions:**

This study is timely, aligning with ongoing national efforts to implement an injury registry. By synthesizing available evidence, we will identify the potential of injury registries to guide the decisions of Brazilian policy makers.

**Trial Registration:**

PROSPERO CRD42023481528; https://www.crd.york.ac.uk/prospero/display_record.php?RecordID=481528

**International Registered Report Identifier (IRRID):**

PRR1-10.2196/55029

## Introduction

Injuries arising from various incidents, such as accidents, falls, drownings, burns, poisonings, and acts of violence directed at oneself or others, represent a global concern. Of the 4.4 million injury-related deaths, an annual toll of 3.16 million is attributed to unintentional injuries, while injuries linked to acts of violence account for 1.25 million casualties annually. Approximately one in 3 fatalities result from road accidents, 1 in 6 from suicides, 1 in 2 from homicides, and 1 in 61 from armed conflicts. According to data from the World Health Organization, globally, approximately 200,000 young individuals aged 10 to 29 lose their lives each year, with homicides ranking as the fourth leading cause of death within this age group [1].

In Brazil, injuries and violence have consistently ranked as the third or fourth leading cause of death among Brazilians, surpassed only by cardiovascular diseases and cancer until 2015, by respiratory diseases between 2016 and 2019, and by COVID-19 during the pandemic. In 2021, there were 149,322 fatalities attributed to injuries in Brazil, equating to approximately 70 deaths per 100,000 Brazilians. Among these causes, homicides prevailed (30.5%), followed by traffic accidents (23.5%), other accidental causes (23.2%), and suicides (10.4%). Studies highlight the significance of injuries among young individuals and males in premature mortality and disabilities, making it a top-priority issue in the country [2,3].

Every year, tens of millions of individuals around the world endure nonfatal injuries, resulting in visits to emergency rooms, acute care facilities, or hospitalizations [1]. In Brazil, in 2021, the number of hospitalizations within the public health system due to injuries was 8.4 times higher than the number of deaths. That year witnessed nearly 600 injury-related hospitalizations per 100,000 Brazilians, totaling 1,247,109 hospitalizations. Among these hospitalizations, falls were the most common (34.6%), followed by other accidental injuries (24.9%), and traffic accidents (18.9%) [4].

The National Policy for the Reduction of Morbidity and Mortality from Accidents and Violence emerged as a response to the challenge of injuries as a public health problem in Brazil, given their magnitude and significance [5]. As a result of this policy, the National Policy for Emergency Care was launched in 2003 [6]. In 2004, the proposal for the creation of the National Network for the Prevention of Violence and Health Promotion was introduced [7]. Two years later, in 2006, the Violence and Accident Surveillance System in Sentinel Services (Viva) was implemented [8]. In 2010, the Life in Traffic Project was initiated, representing an innovative initiative for intersectoral coordination and data integration for the surveillance and intervention in traffic accidents, executed by 54 Brazilian municipalities [9-11].

In 2021, a project under the framework of the Institutional Development Support Program of the Unified Health System (PROADI-SUS), a collaboration between the Brazilian Ministry of Health and the Hospital Israelita Albert Einstein (HIAE), was initiated with the goal of establishing a national injury registry. This initiative aims to facilitate the integration and improve data quality from prehospital and hospital care, as well as various existing health information systems that currently lack interoperability. In addition, it seeks to incorporate data from multiple sectors involved in the topic of injuries and violence, such as firefighters and police. The primary objective of this project is to strengthen health surveillance capacity, providing timely and high-quality information, thus guiding the development and evaluation of prevention strategies and policy making in this field, incorporating data from across the country. Ultimately, these efforts aim to reduce morbidity and mortality associated with injuries [12].

An injury registry is a systematic and standardized database that records detailed information about events and individuals who have sustained injuries, regardless of their severity or whether they result from accidents, falls, violence, or other causes. These databases can encompass a wide range of injuries, from minor ones that may not require hospitalization to more serious injuries that do [13]. Traumatic injuries that require acute care delivered to hospitalized patients, typically resulting from high-impact events such as car accidents, severe falls, or gunshot wounds, are usually included in trauma registries [14]. Both types of registries play important roles in injury prevention, research, and health care management. The primary distinction lies in the scope and severity of injuries each type of registry deals with. They include but are not limited to patient demographic data, the circumstances, characteristics, and outcomes of each injury case, prehospital care, hospital management, and outcomes, with the aim of providing a comprehensive view of the injury landscape in a given region, country, or health care system. These registries often leverage information technology systems to efficiently collect, store, and analyze data. Their primary objectives include enhancing understanding of injury trends, identifying high-risk populations, and evaluating the effectiveness of injury prevention initiatives and trauma care systems. In addition, they allow policy makers to efficiently target resources and implement evidence-based interventions [15-17].

The success of trauma systems has been reported in various localities, with their effectiveness well-established [15,17-19]. Estimates indicate a 15% reduction in the odds of mortality [20,21]. Furthermore, the implementation of trauma registries has been well-documented, and these registries are consistently recognized as essential tools for improving trauma systems and policy making [16,22]. However, a substantial knowledge gap remains regarding the effects of injury registries that include all injuries, irrespective of their severity [22].

Considering the current efforts to implement an injury registry in Brazil, there is an urgent need to establish an evidence base in this context. Therefore, this protocol outlines a systematic review and meta-analysis aimed at investigating the effect of injury registries on policy making and injury outcomes, particularly in terms of reducing hospitalizations and mortality. Hence, the research question guiding this review is “What is the effect of implementation and use of injury registry data on policy making, hospitalization, and mortality?”

## Methods

The development of this protocol adhered to the reporting recommendations outlined in the PRISMA-P (Preferred Reporting Items for Systematic Reviews and Meta-Analyses Protocols) 2015. The checklist is filled in Multimedia Appendix 1 [23]. The systematic review report itself will comply with the PRISMA (Preferred Reporting Items for Systematic Reviews and Meta-Analyses) checklist for 2020 [24].

### Eligibility Criteria

To formulate the eligibility criteria for this systematic review we used the PICOS (Population, Intervention, Comparison, Outcomes, and Study) structure, as shown in Table 1. Included articles must report results or effects related to the implementation and use of data from injury registries, including trauma registries, for at least one of the outcomes described in Table 1. These registries should be established within cities, states, provinces, countries, hospitals, or other well-defined administrative boundaries. Nonrandomized intervention studies or analytical observational studies will be included, such as cohort studies, case-control studies, before-and-after studies, and interrupted time series studies, among others. Descriptive observational studies will be included exclusively for the policy making outcome.

**Table 1:** Eligibility criteria are presented in the PICOS (Population, Intervention, Comparison, Outcomes, and Study) structure.

Acronym	Description	Criteria
P	Population	Cities, states, provinces, countries, hospitals, or other well-defined administrative boundaries with injury surveillance.
I	Intervention	Implementation and use of injury registries, including trauma registries.
C	Comparison	No restrictions on comparators.
O	Outcomes	Policy making (changes and design, including preventive measures, health care improvements, surveillance actions, among others), hospitalization rates or duration, and mortality.
S	Study	Nonrandomized intervention studies or observational studies

Studies will be excluded if they do not provide any description of the injury or trauma registry or are based on sources other than an injury or trauma registry. Furthermore, studies with a specific focus solely on the registry of a particular body part or a specific age group, or those that do not involve human participants, will be excluded. Articles without full-text availability will also be excluded, as well as opinion articles, editorials, letters to the editor, conference abstracts, systematic or literature reviews, and any other studies that lack empirical research regarding the effects of injury registries. Also, studies using decision models or other estimation methods for outcomes will be excluded. The excluded studies will be documented in an attached table within the systematic review.

There will be no restrictions on publication date, country, or language. Studies published in languages other than English, Portuguese, or Spanish will be translated using Google Translate.

### Information Sources

The search will be conducted in the MEDLINE databases through PubMed, Embase, Lilacs through the Virtual Health Library, Scopus, and Web of Science databases. To ensure comprehensive literature coverage, we will also review the reference lists of included studies and relevant systematic or literature reviews identified through the search.

### Search Strategy

The implemented search strategies are presented in Multimedia Appendix 2. The search terms used were defined for the population, intervention, and outcomes, as outlined in the PICOS structure. These terms include controlled vocabulary terms specific to each database, as well as uncontrolled vocabulary terms, synonyms, natural language, related terms, expressions, and truncations, all aimed at broadening the scope of the search.

The strategies were developed by a librarian (ETR) experienced in conducting systematic review searches. First, the MEDLINE strategy was developed with input from the research team and was subsequently reviewed by a specialist (BZdO) with expertise in the research topic. Once the MEDLINE strategy was finalized, it was adapted to the syntax and subject headings of the other databases.

### Data Management

Literature search results will be uploaded to Rayyan (Qatar Computing Research Institute), a free web platform that expedites the elimination of duplicates and the initial screening of abstracts and titles through a semiautomated process [25]. Comments and notes relevant to inclusion and exclusion criteria will be recorded on this platform. After the initial screening, the remaining studies will undergo a full-text review and data extraction, which will be performed using a Microsoft Excel spreadsheet. Excluded studies will also be organized in a Microsoft Excel spreadsheet.

### Selection Process

Adhering to PRISMA recommendations [23], 2 independent reviewers (ACMdS and LESL) will perform the initial screening of titles and abstracts based on the eligibility criteria. The reasons for study exclusions will be documented. Any disagreements not resolved through discussion between the reviewers will be arbitrated by a third reviewer (BZdO).

### Data Collection Process

Using a predefined Microsoft Excel spreadsheet, the same independent reviewers (ACMdS and LESL) will extract the data of interest for the systematic review from each included publication with available data. To ensure consistency among reviewers, calibration exercises will be conducted before starting the review. The data extracted by each researcher will be compared and merged into a single Microsoft Excel spreadsheet. Reviewers will resolve disagreements through discussion, and a third reviewer (BZdO) will adjudicate unresolved disagreements. Authors of the studies will be contacted to address any uncertainties.

### Data Items

The information that will be extracted from each study are publication title, authorship, corresponding author’s contact, publication year, URL, language, summary, objectives, methodology, study type, time horizon, study location, study population, sample size, statistical significance, comparator (if applicable), outcome measures, conflicts of interest, and methodological quality. Concerning the registries, included information will encompass registry type (if injury or trauma registry), registry age, geographical scope, participation and inclusion criteria, number of participating centers, and recorded data. In cases where any data cannot be identified, the authors will be contacted. Additional data items, considered relevant for this systematic review, may also be included if available in eligible studies.

### Outcomes and Prioritization

Recognizing that injury registries are acknowledged for their support in evaluating and designing public policies, thereby enhancing surveillance capabilities and service quality to reduce morbidity and mortality from injuries and violence, the primary outcomes will encompass policy changes and designs, including preventative measures for injuries, health care improvements, surveillance actions, stakeholder partnerships, among others. The secondary outcomes under investigation will involve hospitalization rates or duration, and mortality.

### Risk of Bias in Individual Studies

Risk of bias assessment for each study will be conducted using the ROBINS-I (“Risk Of Bias In Non-randomized Studies-of Interventions”) [26] tool, which is applicable to studies that did not use randomization to allocate interventions, including observational studies. The risk of bias due to confounding, bias in the selection of participants into the study, bias in the classification of interventions, bias due to deviation from intended interventions, bias due to missing data, bias in outcome measurement, and bias in the selection of reported results will be independently assessed by 2 reviewers (ACMdS and LESL). This assessment will categorize the overall risk of bias across the outcomes as low, moderate, serious, critical, or no information. Any unresolved disagreements will be adjudicated through arbitration by a third reviewer (BZdO).

### Data Synthesis

Summaries of study characteristics will be presented in tables and data synthesis will be explored based on outcomes. If studies are sufficiently homogeneous in terms of design and methods, and if 3 or more studies have evaluated the same outcome, a meta-analysis will be conducted using random effects models adapted to the scale of measurement and reported in forest plots. Depending on the available studies, the synthesis method adopted may be changed. Subgroup analyses will be conducted by registry type, whether it is an injury or trauma registry, categorized by injury groups: all injuries, transport accidents, falls, homicides, suicides, and others according to authors’ definitions, as well as by other selected variables of interest. In addition, sensitivity analysis will be carried out, excluding studies of low methodological quality. These analyses will be performed on RStudio software (Posit PBC).

If quantitative synthesis is not appropriate, results will be presented using a standardized narrative synthesis. All relevant findings from the studies will be summarized in the text.

### Meta-Bias

Publication bias, resulting from selective publication or reporting, will be investigated through visual inspection of funnel plots. Statistical tests for assessing symmetry will be explored if 10 or more studies have evaluated the same outcome. These analyses will also be conducted using the RStudio software.

### Confidence in Cumulative Evidence

To evaluate the quality of evidence presented in the meta-analysis, which pertains to the confidence in the effects derived by the set of evidence for a specific outcome, we will adopt the GRADE (Grading of Recommendations Assessment, Development, and Evaluation) approach [27]. Reviewers (ACMdS and LESL) will independently classify evidence as high, moderate, low, or very low quality, considering all factors that determine the reliability of the results presented in the studies. Any disagreement will be settled by consensus or adjudication with a third author (BZdO), if necessary.

## Results

This systematic review will be conducted from November 2023 to June 2024. A preliminary literature search for systematic reviews, both completed and in progress, related to this topic was performed in the Cochrane Database of Systematic Reviews (CDSR) [28] and the Prospective Register of Systematic Reviews (PROSPERO) [29]. No identical review to the proposed study was found. The study was then submitted to PROSPERO on November 10, 2023 (registration number CRD42023481528). The search strategies were finalized, and the bibliographic search commenced on November 6, 2023. The elimination of duplicates and screening of titles and abstracts began in November 2023 as well. In total, 35 studies were retrieved, of which 85 were excluded due to duplication, leaving 50 studies for the selection process. Upon completion, the results of the review will be published in the second half of 2024.

## Discussion

### Principal Findings

This systematic review and meta-analysis is particularly timely, given the current national efforts to implement an injury registry. We strongly believe that our findings will be of crucial importance for anticipating the effects of implementing an injury registry, including trauma registries, on policies and injury outcomes.

Our findings may identify and offer a holistic view of policies and opportunities for intervention that can be established using injury registries. In addition, they may highlight the political partnerships with other sectors involved in the field of injuries and violence, such as firefighters and police, enabling effective intervention strategies based on identified needs. Furthermore, we aim to assess the impact of injury records on reducing hospitalization rate and duration, as well as mortality.

To our knowledge, this is the first systematic review to use evidence intending to map these effects using injury or trauma registry data, preferably with proven causality. Previous research has predominantly focused on this relationship with trauma systems, as evidenced by a meta-analysis that demonstrated a 15% reduction in mortality in favor of the presence of a trauma system [21], as well as by another systematic review that sought to identify which components contributed to its effectiveness [20]. We can also mention another study that exclusively addressed the research dimensions of trauma registry data regarding the formulation of health policies, though it did not encompass all types of injuries [16]. While trauma registries have demonstrated considerable success, particularly in high-income countries, our study addresses a notable gap in the literature by examining the broader implications of injury registries, which encompass a wider spectrum of injuries, regardless of severity.

One of the strengths of our study lies in its transparency and methodological rigor, as it adheres to PRISMA guidelines for systematic reviews and meta-analyses, ensuring the reliability of our findings. However, as with any systematic review, certain limitations need to be anticipated. One potential limitation is the possibility of a shortage of available primary studies on the topic. Furthermore, variability in study designs, data sources, and methodological approaches may pose challenges to data synthesis and drawing conclusive results. This variability can limit the ability to perform robust statistical analyses. The quality or risk of bias of the included studies also needs to be recognized, as this may affect the reported results. In addition, some registries implemented in specific contexts, such as high-income countries, may not be directly comparable to the effects observed in low- and middle-income countries.

To ensure the widespread dissemination of our findings, we plan to publish our results in peer-reviewed journals and present them at relevant congresses and seminars. In addition, we aim to engage policy makers, health care professionals, and stakeholders through targeted dissemination activities, such as interactive workshops and meetings, to facilitate the translation of our findings into practice.

### Conclusion

In conclusion, this systematic review and meta-analysis contribute to the growing body of evidence on the effectiveness of injury or trauma registries in informing policy making and improving health outcomes. By synthesizing available evidence, we will identify the potential of injury registries to enhance health surveillance, reinforce and guide Brazilian policy makers’ decisions, and ultimately reduce the burden of morbidity and mortality associated with injuries in the country. However, further research will be warranted to focus on the impact of injury registries and address the methodological challenges inherent in evaluating their effectiveness causally, even through primary studies.

## Appendix

Multimedia Appendix 1 PRISMA-P (Preferred Reporting Items for Systematic review and Meta-Analysis Protocols) 2015 checklist: recommended items to address in a systematic review protocol*.

Multimedia Appendix 2 Search strategy.

## Abbreviations

CDSR: Cochrane Database of Systematic Reviews
GRADE: Grading of Recommendations Assessment, Development and Evaluation
HIAE: Hospital Israelita Albert Einstein
PICOS: Population, Intervention, Comparison, Outcomes, and Study
PRISMA: Preferred Reporting Items for Systematic Reviews and Meta-Analyses
PRISMA-P: Preferred Reporting Items for Systematic Reviews and Meta-Analyses Protocol
PROADI-SUS: Institutional Development Support Program of the Unified Health System
PROSPERO: Prospective Register of Systematic Reviews
ROBINS-I: Risk Of Bias In Non-randomized Studies-of Interventions
