# Supplementary material for: Effects of Injury Registry Data on Policy Making, Hospitalizations, and Mortality: Protocol for a Systematic Review and Meta-Analysis
Source: JMIR Res Protoc. 2024 Oct 30;13:e55029. doi: 10.2196/55029 (PMC11561427; doi:10.2196/55029)
Supplement: Multimedia Appendix 2 [file resprot_v13i1e55029_app2.docx]

Search strategy

| **Database** | **Search strategy** |
| --- | --- |
| PubMed | ("wounds and injuries/epidemiology"[MeSH Terms] OR "wounds and injuries/mortality"[MeSH Terms] OR "wounds and injuries/prevention and control"[MeSH Terms] OR (("wound*"[Text Word] OR "injur*"[Text Word] OR "trauma*"[Text Word] OR "external cause*"[Text Word]) AND ("epidemiology"[Text Word] OR "mortalit*"[Text Word] OR "prevention and control"[Text Word]))) AND  ("trauma registr*"[Text Word] OR "trauma data*"[Text Word] OR "injury registr*"[Text Word] OR "injury data*"[Text Word])  AND  ("Policy Making"[MeSH Terms] OR "Policy Making"[Text Word] OR "Health Policy"[MeSH Terms] OR "health care polic*"[Text Word] OR "health polic*"[Text Word] OR "outcome assessment, health care"[MeSH Terms] OR "outcomes assessment*"[Text Word] OR "Outcome study"[Text Word] OR "health outcome*"[Text Word] OR "HIA"[Text Word] OR "impact*"[Text Word] OR "effect*"[Text Word]) |
| Embase | (('injury'/mj OR 'accidental injury'/mj OR 'wound*':ti,ab,kw OR 'external cause*':ti,ab,kw OR 'injur*':ti,ab,kw OR 'trauma*':ti,ab,kw) AND ('epidemiology'/exp OR 'mortality rate'/exp OR 'prevention and control'/exp)) AND ('trauma data*':ti,ab,kw OR 'trauma registr*':ti,ab,kw OR 'registr* of trauma':ti,ab,kw OR 'injur* registr*':ti,ab,kw OR 'injur* data*':ti,ab,kw OR 'registr* of injur*':ti,ab,kw) AND ('health care policy'/mj OR 'public policy'/mj OR 'outcome assessment'/exp OR 'health impact assessment'/exp OR 'impact':ti,ab,kw OR 'effect*':ti,ab,kw OR 'hia':ti,ab,kw) |
| Lilacs | ((mh:(Wounds and Injuries)) OR (mh:(Ferimentos e Lesões)) OR (mh:(Heridas y Lesiones)) OR (wound*) OR (Injur*) OR (Trauma*) OR (mh:(External Causes)) OR (Ferida*) OR (Ferimento*) OR (Lesão) OR (Lesões) OR (Herida*) OR (Lesión) OR (Lesiones) OR (mh:(Causas Externas))) AND ((sh:(epidemiologia)) OR (sh:(mortalidade)) OR (sh:(prevenção & controle))) AND ((“trauma registry”) OR (“trauma registries”) OR (“trauma databank”) OR (“trauma database”) OR (“trauma dataset”) OR (“trauma data bank”) OR (“trauma data base”) OR (“trauma data set”) OR (“injury registry”) OR (“injury registries”) OR (“injury databank”) OR (“injury database”) OR (“injury dataset”) OR (“injury data bank”) OR (“injury data base”) OR (“injury data set”) OR (“registro de trauma”)) AND ((mh:(Policy Making)) OR (mh:(Formulação de Políticas)) OR (mh:(Formulación de Políticas)) OR (Policy Making) OR (mh:(Health Policy)) OR ("Health Public Policy") OR ("Health Public Policies") OR (mh:(Política de Saúde)) OR ("Política Pública de Saúde") OR ("Políticas Públicas de Saúde") OR (mh:(Política de Salud)) OR ("Políticas Públicas de Salud") OR ("Políticas Públicas Saludables") OR (mh:(Outcome Assessment, Health Care)) OR ("Outcomes Assessment") OR ("Outcomes Study") OR (mh:(Avaliação de Resultados em Cuidados de Saúde)) OR ("Avaliação de Resultados") OR ("Estudos de Resultados") OR (mh:(Evaluación de Resultado en la Atención de Salud)) OR ("Evaluación de los Resultados") OR (HIA) OR (impact*) OR (effect*)) |
| Scopus | ( ( TITLE-ABS-KEY ( "Wounds and Injuries" ) OR TITLE-ABS-KEY ( "Wound*" ) OR TITLE-ABS-KEY ( "Injur*" ) OR TITLE-ABS-KEY ( "Trauma*" ) OR TITLE-ABS-KEY ( "external cause*" ) ) AND ( TITLE-ABS-KEY ( "epidemiology" ) OR TITLE-ABS-KEY ( " Population Health" ) OR TITLE-ABS-KEY ( "mortalit*" ) OR TITLE-ABS-KEY ( "prevention and control" ) ) ) AND ( TITLE-ABS-KEY ( "trauma data*" ) OR TITLE-ABS-KEY ( "trauma registr*" ) OR TITLE-ABS-KEY ( "injury data*" ) OR TITLE-ABS-KEY ( "injury registr*" ) ) AND ( TITLE-ABS-KEY ( "Policy making" ) OR TITLE-ABS-KEY ( "Health Polic*" ) OR TITLE-ABS-KEY ( "Health care polic*" ) OR TITLE-ABS-KEY ( "Health outcome*" ) OR TITLE-ABS-KEY ( "Outcomes assessments" ) OR TITLE-ABS-KEY ( "Outcome study" ) OR TITLE-ABS-KEY ( "HIA" ) OR TITLE-ABS-KEY ( "Impact*" ) OR TITLE-ABS-KEY ( "Effect*" ) ) |
| Web of Science | (TS=(“Wounds and Injuries" OR "Wound*" OR "Injur*" OR "Trauma*" OR "external cause*") AND TS=("epidemiology" OR "mortalit*" OR "prevention and control")) AND (TS= ( "trauma registr*" OR "trauma data*" OR "registr* of trauma*" OR "injur* data*" OR "injury registr*" OR "registr* of injur*")) AND (TS=("Policy making" OR "Health Polic*" OR "Health care polic*" OR "Health outcome*" OR "Outcomes assessments" "Outcome study" OR "HIA" OR "Impact*" OR "Effect*")) |
